# Implementation of next generation sequencing into pediatric hematology-oncology practice: moving beyond actionable alterations

**DOI:** 10.1186/s13073-016-0389-6

**Published:** 2016-12-23

**Authors:** Jennifer A. Oberg, Julia L. Glade Bender, Maria Luisa Sulis, Danielle Pendrick, Anthony N. Sireci, Susan J. Hsiao, Andrew T. Turk, Filemon S. Dela Cruz, Hanina Hibshoosh, Helen Remotti, Rebecca J. Zylber, Jiuhong Pang, Daniel Diolaiti, Carrie Koval, Stuart J. Andrews, James H. Garvin, Darrell J. Yamashiro, Wendy K. Chung, Stephen G. Emerson, Peter L. Nagy, Mahesh M. Mansukhani, Andrew L. Kung

**Affiliations:** 1 Department of Pediatrics, Columbia University Medical Center, New York, NY 10032 USA; 2 Department of Pathology and Cell Biology, Columbia University Medical Center, New York, NY 10032 USA; 3 Department of Clinical Genetics, Columbia University Medical Center, New York, NY 10032 USA; 4 Department of Medicine, Columbia University Medical Center, New York, NY 10032 USA; 5 Department of Microbiology and Immunology, Columbia University Medical Center, New York, NY 10032 USA; 6 Herbert Irving Comprehensive Cancer Center, Columbia University Medical Center, New York, NY 10032 USA; 7 Present address: Memorial Sloan Kettering Cancer Center, New York, NY 10065 USA; 8 Present address: MNG Laboratories, 5424 Glenridge Drive, Atlanta, GA 30342 USA

**Keywords:** Whole exome sequencing, RNA sequencing, Precision medicine, Pediatric oncology

## Abstract

**Background:**

Molecular characterization has the potential to advance the management of pediatric cancer and high-risk hematologic disease. The clinical integration of genome sequencing into standard clinical practice has been limited and the potential utility of genome sequencing to identify clinically impactful information beyond targetable alterations has been underestimated.

**Methods:**

The Precision in Pediatric Sequencing (PIPseq) Program at Columbia University Medical Center instituted prospective clinical next generation sequencing (NGS) for pediatric cancer and hematologic disorders at risk for treatment failure. We performed cancer whole exome sequencing (WES) of patient-matched tumor-normal samples and RNA sequencing (RNA-seq) of tumor to identify sequence variants, fusion transcripts, relative gene expression, and copy number variation (CNV). A directed cancer gene panel assay was used when sample adequacy was a concern. Constitutional WES of patients and parents was performed when a constitutionally encoded disease was suspected. Results were initially reviewed by a molecular pathologist and subsequently by a multi-disciplinary molecular tumor board. Clinical reports were issued to the ordering physician and posted to the patient’s electronic medical record.

**Results:**

NGS was performed on tumor and/or normal tissue from 101 high-risk pediatric patients. Potentially actionable alterations were identified in 38% of patients, of which only 16% subsequently received matched therapy. In an additional 38% of patients, the genomic data provided clinically relevant information of diagnostic, prognostic, or pharmacogenomic significance. RNA-seq was clinically impactful in 37/65 patients (57%) providing diagnostic and/or prognostic information for 17 patients (26%) and identified therapeutic targets in 15 patients (23%). Known or likely pathogenic germline alterations were discovered in 18/90 patients (20%) with 14% having germline alternations in cancer predisposition genes. American College of Medical Genetics (ACMG) secondary findings were identified in six patients.

**Conclusions:**

Our results demonstrate the feasibility of incorporating clinical NGS into pediatric hematology-oncology practice. Beyond the identification of actionable alterations, the ability to avoid ineffective/inappropriate therapies, make a definitive diagnosis, and identify pharmacogenomic modifiers is clinically impactful. Taking a more inclusive view of potential clinical utility, 66% of cases tested through our program had clinically impactful findings and samples interrogated with both WES and RNA-seq resulted in data that impacted clinical decisions in 75% of cases.

**Electronic supplementary material:**

The online version of this article (doi:10.1186/s13073-016-0389-6) contains supplementary material, which is available to authorized users.

## Background

The outcomes for children with cancer have steadily improved to the present time when more than 80% of all pediatric oncology patients are cured [1]. Nonetheless, cancer remains the leading cause of disease-related death in children. Moreover, this success has come at a price; two-thirds of all survivors have some long-term sequelae attributable to their treatment [2]. Together, the requirement to further improve existing outcomes and to decrease toxicity underscores the need for the current national initiative in precision medicine to include pediatric oncology patients.

Many of the advances in pediatric oncology have resulted from the implementation of risk-stratified treatment strategies that incorporate histological, anatomical, and molecular prognostic and predictive determinants into the choice of therapies for individual patients [3]. Changes in ploidy, chromosomal segmental changes, and specific gene alterations are routinely utilized to guide treatment intensity in pediatric oncology [4]. Therefore, the tenants of precision medicine are intrinsic to the practice of pediatric oncology.

Recent advances in massively parallel sequencing allow for more comprehensive approaches to determine the abnormalities contributing to tumorigenesis. Initial implementation of next generation sequencing (NGS) technologies focused on the identification of actionable alterations, with estimates in the range of 5% to nearly 100% depending on disease histology and evolving definitions of “actionable” [5–15]. The utility of these technologies, however, extends well beyond the identification of actionable alterations and determining the value of these technologies should be more inclusive and consider the broad clinical impact of testing.

In 2014, we implemented a clinical NGS platform for pediatric oncology patients. The Precision in Pediatric Sequencing (PIPseq) Program utilizes NGS of tumor and germline in a CLIA-certified (Clinical Laboratory Improvement Amendments of 1988) environment and includes interrogation of both DNA and RNA. We conducted a retrospective review of our first 101 consecutively sequenced patients utilizing the PIPseq pipeline and report here our experience with integrating clinical NGS into pediatric hematology-oncology practice and describe the broad clinical utility of genomically informed cancer medicine.

## Methods

### The PIPseq pipeline

To achieve more comprehensive genome-level analysis in our pediatric oncology patients, we utilized three CLIA-certified, CAP (College of American Pathologists), and New York State Department of Health approved assays. When possible, we utilized a cancer whole exome sequencing test (cWES) comprising WES of tumor and normal tissue (buccal swab or peripheral blood) and RNA sequencing (RNA-seq) of tumor tissue. This assay was optimized for fresh or frozen specimens. When sample adequacy was a concern, we also utilized a directed cancer gene panel assay which sequenced 467 cancer-associated genes and was optimized for use with formalin fixed paraffin embedded (FFPE) material (Columbia Comprehensive Cancer Panel, CCCP). If a constitutionally encoded disease was suspected (e.g. familial hemophagocytic lymphohistiocytosis), we performed constitutional WES from the patient and both parents (trio) when available.

Tissue for sequencing was obtained either from archived blocks (FFPE) or frozen tissue blocks from the Department of Pathology. DNA and RNA extraction and sequencing were performed in a CLIA-certified laboratory. Variant calls were independently made on tumor and germline material and somatic variants determined by subtraction. Copy number variation (CNV) was determined from the WES data, fusion transcripts were identified from the RNA-seq data, and relative gene expression was determined by comparison to a model built from 124 transcriptomes. A mix of tissues was used to generate the model including normal white blood cells, lung, liver, brain, glioma, and cell lines.

After initial review by a molecular pathologist, all results were reviewed in a multi-disciplinary molecular tumor board. Participants included representation by molecular pathology, pediatric oncology, cytogenetics, medical genetics, and cancer biology. For each patient, a report was issued containing variant calls, CNV, fusions, and overexpressed genes. Variants were assigned a tier based on disease-association and separately a tier based on level of evidence for clinical actionability (described below). Reports were delivered to ordering oncologists and posted to the electronic medical record (EMR) in accordance with patient opt-in/opt-out preferences selected at the time of informed consent.

### Patients and informed consent for clinical sequencing

Between January 2014 and April 2016, NGS was performed on tumor and/or normal tissue from 101 high-risk patients by the Laboratory of Personalized Genomic Medicine at Columbia University Medical Center (CUMC). This represented approximately 32% of the total patients in our clinical practice. High-risk patients were defined as those having a prognosis of <50% overall survival at 5 years, outlier clinical phenotype, rare cancer without standard of care therapy, suspected cancer predisposition, or relapsed disease. A request for constitutional WES, cWES and RNA-seq, or targeted cancer panel testing was made at the discretion of the referring oncologist in consultation with the PIPseq team [16].

Participants signed consent for WES or cWES either as part of an Institutional Review Board (IRB)-approved protocol (IRB nos. AAAB7109, AAAJ5811) or they signed the clinical consent (http://pathology.columbia.edu/diagnostic/PGM/oncologytests.html). Written consent for clinical WES and cWES testing was obtained after the risks and benefits had been explained to the patient and/or caregiver, which include the potential disclosure of medically actionable secondary findings, defined as germline disease-causing mutations unrelated to the condition for which sequencing was being performed. Patients could opt in or opt out of the following: learning secondary findings and/or having these results appear in the EMR; having their samples and/or data stored for future research, both with or without identifiers; and future contact. Results not reported included carrier status, variants of uncertain significance (VOUS) in secondary findings except as related to cancer, and mutations related to adult-onset conditions for which the genetic link is either unclear or for which no known intervention is of proven benefit (e.g. Alzheimer’s disease). IRB approval was obtained for this retrospective analysis of de-identified patient and clinical genomics data (IRB nos. AAAP1200 and AAAQ8170).

### Clinical sequencing

Testing required at least 200 ng of DNA for WES, at least 50 ng of DNA for targeted DNA sequencing, and at least 3000 ng of RNA for transcriptome analysis (Additional file 1: DNA and RNA extraction). The entire assay was a CLIA-certified assay. The laboratory-developed test used general purpose reagents and the Agilent WES ver.5 + UTR baits. Specifically, WES was performed using the Agilent SureSelectXT All Exon V5 + UTRs capture kit for library generation and sequenced on the HiSeq2500 using paired-end 125 cycle × 2 sequencing (two tumors, two normal and two transcriptomes, pooled together and run in two lanes). Targeted DNA sequencing was performed on a 5.59 Mb Custom Agilent SureSelectXT library, targeting 467 genes, and sequenced on a HiSeq2500 using paired-end 125 cycle × 2 sequencing (seven samples per lane). RNA was sequenced using the TruSeq Stranded Total RNA LT Sample Prep Kit with 125 cycles × 2 paired-end sequencing on the HiSeq2500.

### Sequencing analysis

DNA sequencing reads were de-multiplexed and converted to fastq files using CASAVA from Illumina. Mapping and variant calling of tumor and normal samples was performed using NextGene (v.2.3.4; Softgenetics, State College, PA, USA), which uses a modified Burrows-Wheeler transform (BWT) alignment method. Sequences were mapped to GRCh37 (“hg19”), retaining reads with a median quality score of 20 or higher, with no more than three ambiguous bases, a minimum number of 25 called bases per read, and trimming reads when three consecutive reads fell below a quality score of 16. Alignment and variant calling was performed using paired-end reads with a minimum of 10 reads, at least three variant reads, and a minimum variant allelic fraction of 10% for tumor and 5% for normal was required to call a variant. The variant calling module was set to “detect large indels.” The variant calling algorithm showed a 99.6% agreement with single nucleotide polymorphisms on an oligonucleotide microarray and over 96% sensitivity in inter-laboratory comparison and a 96% detection rate for heterozygous variants in a 40/60% mixture of samples. For small indels, the lab detected 93% of all variants detected by another laboratory in inter-laboratory comparison, with the greatest disagreement in insertions greater than 10 bp.

Variants were subject to filtering. In normal DNA, variants were passed through a “reference range filter” of cancer predisposition genes, genes relevant to pharmacogenomics, and variants relevant to patient care; a “reportable range filter” which includes COSMIC (cosmic70 provided by Annovar) variants in the patient’s mutation report file and variants in genes recommended by the American College of Medical Genetics (ACMG) for the reporting of secondary findings [17]; as well as a frequency filter, which includes variants whose minor allele frequency in the 1000 Genomes (phase 1, version 3, release date 23 November 2010) is less than 1%. Somatic mutations in the tumor were identified by subtracting all variants called in normal tissue (output at minor allelic fraction of ≥5%) from variants called in the tumor (output at a minor allelic fraction of ≥10%). The approach maximized the number of variants output to minimize the likelihood of filtering out actionable mutations prior to molecular tumor board discussion (Additional file 1: Supplementary Methods; Somatic Variant Calling Strategy).

Variants in the tumor were further characterized as homozygous, compound heterozygous, somatic, and “disruptive” (loss of function, namely, nonsense, frame-shift, or splice site). Spreadsheets with the various categories were presented to molecular pathologists for review. Quality statistics for WES and cWES are presented in Additional file 2: Table S1. Targeted DNA sequencing was performed to an average 500X depth and analyzed as above. All DNA sequencing results were manually reviewed by molecular pathologists to prioritize variants for presentation at the multi-disciplinary tumor board and subsequent reporting of consensus variants. For mutation statistics, the list of “tumor-specific” variants obtained by comparison of vcfs was filtered for variants with at least 30X coverage in tumor and either a “quality score” ≥20 or a variant allelic fraction ≥25% in the tumor.

#### Copy number variation

CNV was identified using EXCAVATOR (v.2.2; https://sourceforge.net/projects/excavatortool) software [18]. For samples with greater than 95% of targeted nucleotides present at least 10X in the reference normal and at least 90% covered 30X in the corresponding tumor sample, EXCAVATOR was run with parameters chosen for moderate sensitivity (assuming a tumor percentage of 0.8) and cutoff for loss set to a log2 ratio of –0.2. In addition, all high quality heterozygous variants with variant allelic fractions (VAFs) in the range of 45–55% and 90–100% in the normal sample were output. The allelic ratio at these genomic coordinates in the tumor was also output for viewing on the integrated genomic viewer to allow identification of copy number neutral loss of heterozygosity (LOH) and to support the CNVs identified by EXCAVATOR. The laboratory detected all chromosome arm changes seen on karyotyping, losses of 26 Mb and greater seen on array CGH, and reproducibly identified all CNVs that involved at least ten exons at 40% tumor fraction (Additional file 1: Supplementary Methods).

#### Transcriptome analysis

For transcriptome analysis, fastq files from CASAVA were filtered for ribosomal RNA (rRNA) using SortMeRNA (v.1.7; http://bioinfo.lifl.fr/RNA/sortmerna/) and trimmed to remove poor-quality tails using TrimGalore (v.0.2.7; http://www.bioinformatics.babraham.ac.uk/projects/trim_galore/) with settings to exclude reads of quality score <20 and read length <20. Remaining reads were mapped to GRCh37 (hg19) using Tuxedo Suite [19, 20] consisting of TopHat2 (v.2.0.8), BOWTIE2 (v.2.1.0), and CUFFLINKS (v.2.1.1). Non-uniquely mapped reads were excluded before estimation of fragments per kilobase per million reads (FPKMs) by CUFFLINKS. Mutation calling was performed using NextGene software. At least 50 million uniquely mapped reads were required with less than 5% DNA contamination. In addition, unmapped reads were analyzed using “FusionMap” (v.01/01/2015) to generate a list of fusions for review by molecular pathologists [21]. To identify alterations in gene expression, the median FPKMs of 8000 housekeeping genes were used as reference [22] and the relative expression of each gene was compared to 124 normal transcriptomes from various tissues (13 blood, 20 liver, 24 kidney, 17 lung, and 50 brain) (Additional file 1: Supplementary Methods).

### Data interpretation and reporting

Interpretation of clinical WES, RNA-seq, and CNV was conducted via a molecular tumor board with multi-disciplinary representation from pediatric oncology, pathology, cancer biology, molecular and clinical genetics, and bioinformatics. Following the tumor board, approximately 60 days after testing request, a tiered report was generated for clinical samples by pathology, sent to the referring physician and posted to the EMR according to the opt-in/opt-out selections of the patient consent. Only variants with good normal coverage (generally at least 30X) were detected on multiple independent fragments and were not excluded as likely benign were reported. For clinical testing, the report included variants that were justified by the literature as driver mutations (e.g. well characterized hot spot mutations); unambiguous loss of function mutations in tumor suppressor genes (i.e. nonsense or frame-shift mutations that resulted in loss of functional domains); mutations with published laboratory data documenting gain or loss of function in oncogenes and tumor suppressor genes, respectively; and previously reported fusions or fusions that were expected to have the same effect as previously reported fusions involving one of the partner genes. Certain exceptions for clinical testing were made. For example, if a variant was likely a strong driver (e.g. a known activating mutation of an oncogene) but had low coverage in the normal or appeared low-quality on review, the molecular pathologist still considered it but required independent confirmation by an orthogonal method prior to reporting.

The final clinical cWES report included: known tumor type-specific actionable somatic mutations (Tier 1); somatic mutations in targetable pathways, actionable somatic mutations in other tumor types, somatic mutations in well-established cancer genes (Tier 2); other somatic mutations in cancer genes (Tier 3); and somatic VOUS (Tier 4). Reporting of germline findings included: known pathogenic secondary ACMG variants [17]; secondary non-ACMG variants and selected VOUS in known cancer genes with commentary; and known variants that influence pharmacogenomics. Reports further included translocations, significantly overexpressed genes, and segmental CNV. A sample cWES report is presented in Additional file 3. Accession number for all genes and fusions referenced in the paper are reported in Additional file 2: Table S2. Datasets are available through the cBioPortal for Cancer Genomics (http://cbioportal.org) [23, 24].

Clinical utility, defined as the ability of a molecular test result to provide information related to the care of the patient and his/her family members to diagnose, monitor, prognosticate or predict disease progression, and inform treatment [25], was used to evaluate the potential impact of findings from clinical sequencing. “Clinical impact” and “clinically impactful” are broad terms used throughout this paper to refer to any molecular test result that, when integrated with a patient’s history, symptoms, and other clinical findings, informed the medical team’s assessment or management of the patient. These clinically meaningful results were subcategorized into the following five categories to evaluate the potential clinical utility of tumor and germline alterations: (1) diagnostic; (2) prognostic; (3) identification of a therapeutic target; (4) other clinically impactful information, including pharmacogenomics or findings that resulted in a significant refinement of a therapeutic plan (e.g. choice of donor or withdrawal of recommendation for bone marrow transplantation); and (5) recommendations for health maintenance interventions or genetic counseling for the patient and other at-risk family members. Genetic alterations were considered targetable if: (1) an FDA approved drug or experimental drug was available that inhibited the target directly or inhibited its downstream signaling pathway; or (2) there was preclinical evidence to support efficient targeting of the aberrant function of the mutated gene and/or potential clinical benefit; and (3) there was some age-appropriate information on dosing. Targetable somatic mutations were further categorized using a five-tier system previously described by Wagle et al. [26] and Harris et al. [15]. This sub-tiering system uses the strength of preclinical and clinical data as evidence to support the potential clinical benefit of targeting an altered gene with a specific therapeutic agent.

## Results

### Patients

**Fig. 1:**
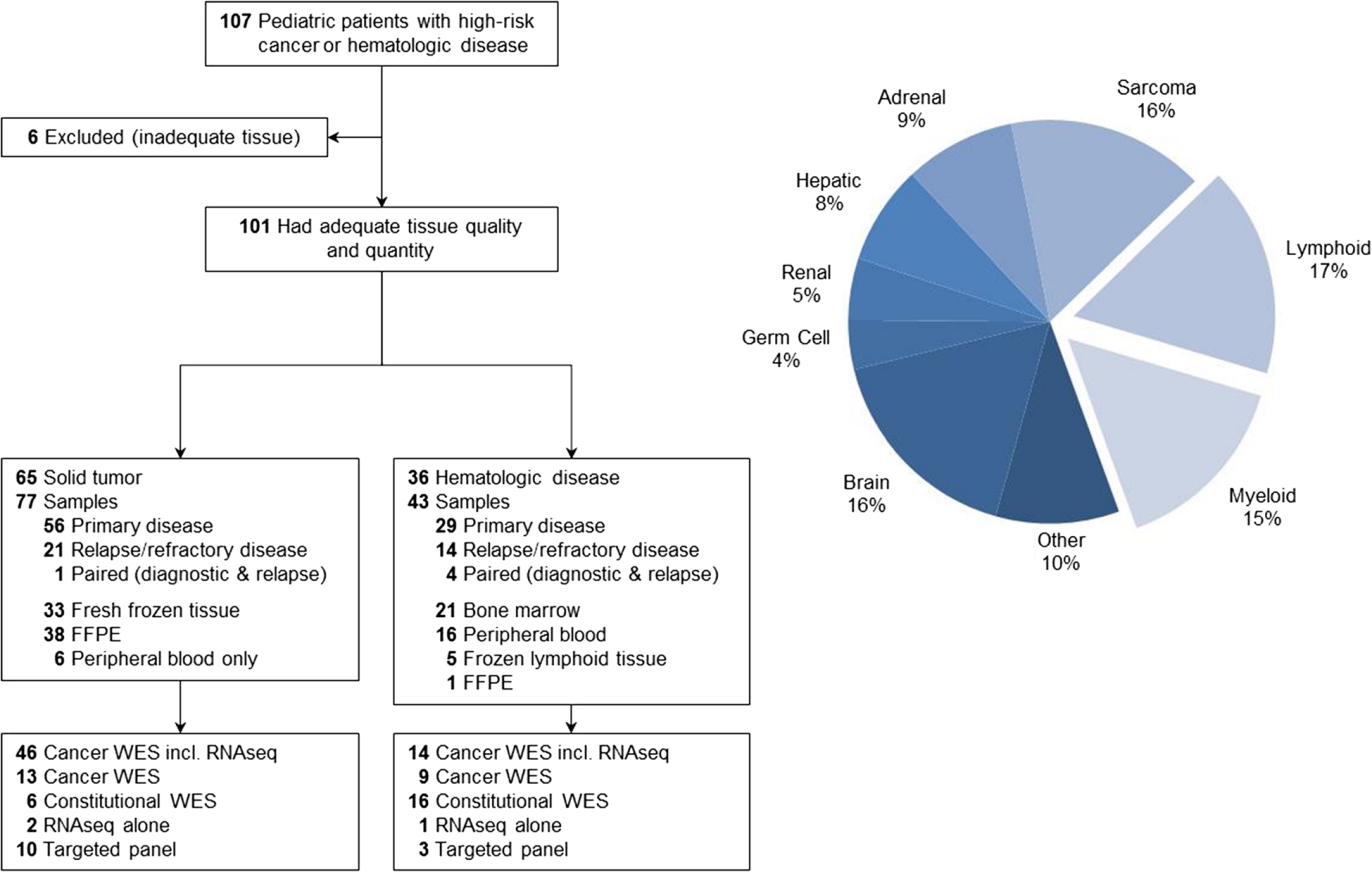
PIPseq overview. An overview of the PIPseq patients sequenced is presented on the *left* and a *pie chart* showing the distribution of diagnostic categories on the *right*

Demographic and clinical characteristics are presented in Table 1 and Fig. 1. Molecular characterization was performed on 120 samples (85, primary disease; 35, relapse/refractory disease) from 101 consecutive cases (mean age, 9.3 years; median age, 8.0 years; range, 2 weeks – 26 years). Patients aged over 18 years in this cohort were initially diagnosed with a pediatric disease under the age of 18 years. Testing included: full cWES (tumor, germline, and transcriptome; *n* = 63); cWES without transcriptome (*n* = 19); transcriptome only (*n* = 3); targeted tumor panel sequencing (*n* = 13); and constitutional WES (proband and parental blood) (*n* = 22). For constitutional WES, trios (proband and both parents) were performed in 18/22 cases, 3/22 cases only had one parent available for testing, and in one case only the proband was tested post-mortem. Eighty-four patients underwent single platform testing, while multiple sequencing platforms were used for 17 cases (36 samples). Cases were predominantly pediatric patients with solid tumors (64%) (Fig. 1; Additional file 2: Table S3). Sarcoma (*n* = 17) was the most common diagnostic sub-category followed by brain tumors (*n* = 16). Patients with lymphoid disease (*n* = 17) comprised the majority of hematological conditions (Fig. 1; Additional file 2: Table S3).

**Table 1:** Patient and sample characteristics (n = 101)

	n (%)
Diagnostic category	
Solid tumors	65 (64)
Hematologic conditions	36 (36)
Gender	
Male	60 (59)
Female	41 (41)
Age (years)	
0–5	35 (34)
6–12	30 (30)
13–17	20 (20)
≥18	16 (16)
Samples tested (n = 120)	
Primary disease	85 (71)
Relapse/refractory	35 (29)
Platform (n = 120)	
Cancer WES with transcriptome	63 (53)
Cancer WES	19 (16)
Constitutional WES	22 (18)
Transcriptome only	3 (2)
Targeted panel (467 genes)	13 (11)
Normal tissue source (n = 104)	
Blood	78 (74)
Buccal swab	23 (23)
Unaffected tissue	3 (3)

*WES* whole-exome sequencing

### Informed consent, cost, and reimbursement

All patients were consented to genomic analysis either through a research consent or clinical WES consent. Of the 101 cases, 67 were consented using the clinical cWES consent. Only four (6%) opted out of learning secondary findings and 21 (31%) opted out of having secondary findings in their medical record. All patients consented to have leftover samples stored. Only a single patient (2%) opted out of future contact (Additional file 2: Table S4).

As part of clinical implementation, we assessed the cost of cWES and RNA-seq and the reimbursement landscape. The total cost per case was calculated by summing the total variable cost (reagent cost, pathologist time) with the fixed cost per case (annual machine cost, annual maintenance, tech labor cost, informatics cost, space for NGS hardware, server time, NGS analysis lease, and data storage). The estimated cost of WES (tumor/normal) was $4459 and the cost of RNA-seq was $1764. These estimates do not include administrative overhead and billing for services.

The time to receiving final reimbursement decisions from third-party payers ranged between 6 months to 1 year. To date, we have received a decision for 56 patients with 45/56 (80%) receiving partial reimbursement. The average reimbursement by carrier type was as follows: commercial, $2747 (range, $770–6917); managed government plans, $2918 (range, $750–4555); and $0 from government plans. Patients and their families were not charged for sequencing or analysis.

### Genomic alterations in pediatric solid tumors and hematologic disorders

**Fig. 2:**
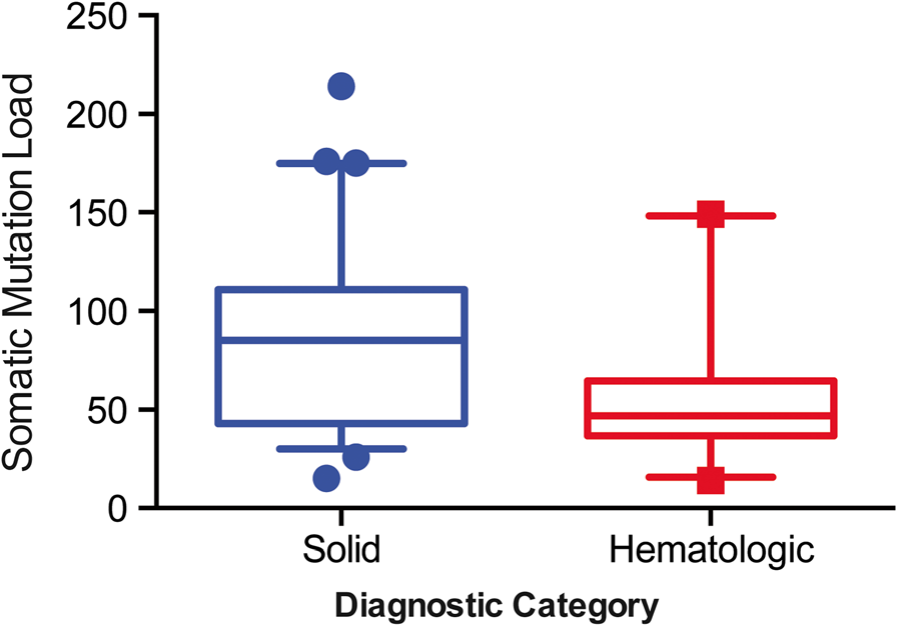
Somatic mutation load by diagnostic category. *Box plots* comparing overall somatic mutation rates across solid tumors and hematologic conditions detected by NGS. The *top* and *bottom* ends of the *boxes* represent the 25th and 75th percentile values, respectively, and the segment in the *middle* is the median. The *top* and *bottom* extremes of the bars extend to the minimum and maximum values. The *box plot* depicts the total mutation load excluding four outliers (one solid tumor and three hematologic). See Additional file 4: Figure S1 for inclusive dataset with outliers. The total mutational load (prior to filtering or orthogonal validation) for solid tumors was 4972 variants (mean, 84.3; SD, 43.9; median, 85; range, 15–214) and for hematologic conditions was 1478 variants (mean, 56.85; SD, 34.9; median, 47; range, 14–149)

**Fig. 3:**
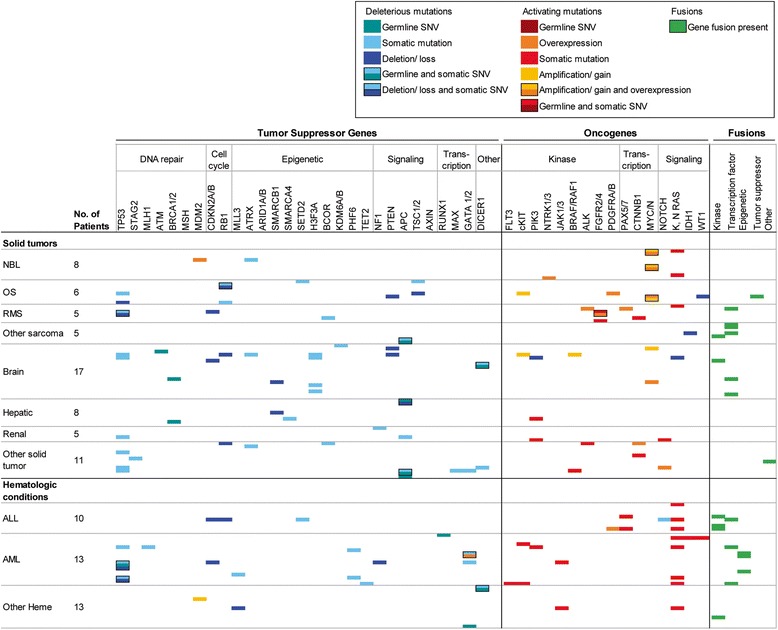
Summary of informative results from the PIPseq program. A matrix representation of findings with biological significance from the sequencing results are presented. Data are derived from all 101 patients that underwent WES of tumor-normal sample pairs, exome sequencing of germline DNA, transcriptome analysis of tumor, CNV of tumor, and targeted panel sequencing of tumor only. Deleterious mutations were loss of function mutations and activating mutations refers to recurrent, previously reported activating mutations in oncogenes or variants with published in vitro evidence as being activating

Over 150-fold and 500-fold average coverage was achieved by WES and targeted capture sequencing, respectively with >98% of the coding sequences having at least tenfold coverage. The mean mutational load across patients was 216.9 variants (SD = 829.3, median = 69), with a higher mean mutational load in solid tumors compared to hematologic malignancies (Fig. 2; Additional file 4: Figure S1). Genomic aberrations were reported in 92/101 patients (91%). After filtering, a total of 180 mutations (Additional file 2: Table S5) and 20 fusions were reported, 110 (including 10 fusions) from solid tumor samples (mean number of aberrations per sample, 2.91; median, 2.00; range, 1–6) and 90 (including 10 fusions) from hematologic samples (mean number of aberrations per sample, 5.2; median, 4.0; range, 1–12). The most commonly mutated gene was *TP53* (*n* = 9, 9%) in solid tumor samples and RAS pathway constituents (*NRAS:* *n* = 5, 5%; *KRAS:* *n* = 3, 3%) in hematologic samples (Fig. 3). In addition, significant changes in the pattern of genetic alterations were noted on serial sequencing of samples from individual patients at different time points during their therapy, reflecting clonal evolution. Awareness of these changes is important for selecting an appropriate targeted therapy and assessing response to therapy.

### Evaluating potential clinical utility and targetable alterations

A genetic variant was considered targetable if: (1) an FDA-approved drug or experimental drug was available that inhibited the target directly or inhibited its downstream signaling pathway; or 2) there was preclinical evidence to support efficient targeting of the aberrant function of the mutated gene and/or potential clinical benefit; and 3) there was some age-appropriate information on dosing. Consistent with the published recommendations from the Association for Molecular Pathology [25], we evaluated clinical utility based on “the ability of a test result to provide information to the patient, physician, and payer related to the care of the patient and his/her family members to diagnose, monitor, prognosticate, or predict disease progression, and to inform treatment and reproductive decisions.”

### Targetable somatic genomic alterations

Overall, 38/101 patients (38%) had at least one potentially targetable genomic alteration (Table 2). Specifically, 21/65 patients (32%) with solid tumors and 17/36 (47%) patients with hematologic conditions carried targetable alterations. Matched therapy based on genomic findings was received in 6/38 patients (16%).

**Table 2:** Sub-classification of potentially targetable somatic mutations for treatment planning

PIP ID	Diagnosis	Target alteration	Mutation (change)	Potential target therapy
Tier 1 (Data demonstrating benefit- same tumor type, same gene)				
14-85546	CML	*BCR-ABL1*	Fusion	TKI
13-45348^a^	AML	*IDH1*	c.394C > T (p.R132C)	IDH Inhibitor
14-24794^a^	AML	*c-KIT; TET2; FLT3*	c.2446G > C (p.D816H); c.3663delT (p.C1221Wfs); c.2505 T > G (p.D835E)	TKI; Hypomethylating agent; TKI
14-53198	AML	*TET2*	c.1156G > T (p.V386L)	Hypomethylating agent
13-77086	AML	*c-KIT*	c.1965 T > G (p.Asn655Lys)	TKI^b^
Tier 2 (Data demonstrating benefit- different tumor type, same gene)				
15-18928	Hepatic rhabdoid tumor	*SMARCA4*	c.3574C > T (p.R1192C)	EZH2 Inhibitor
14-13487	Osteosarcoma	*TSC1*	c.2503-1G > C (p.?)	mTOR Inhibitor
14-47205	Nephroblastomatosis	*PIK3CA*	c.1035 T > A (p.N345K)	PI3K/AKT/mTOR Inhibitor
15-40141	Pleomorphic xanthoastrocytoma	*TMEM106B-BRAF*	Fusion	MEK Inhibitor
Tier 3 (Published, presented, in press pre-clinical data demonstrating benefit- same tumor type, same gene)				
15-64793	ALL	*FOXP1-ABL1*	Fusion	TKI^b^
15-26188	ALL	*NUP214-ABL1*	Fusion	TKI^b^
15-79700	ALL	*NRAS*	c.183A > T (p.Q61H)	MEK Inhibitor
14-20062	ALL	*KRAS*	c.183A > T (p.Q61H)	MEK Inhibitor
14-24794^a^	AML	*NRAS*	c.183A > C (p.Q61H)	MEK Inhibitor
15-29224	AML	*JAK3*	c.1718C > T (p.A573V)	JAK Inhibitor
13-45348^a^	AML	*NRAS*	c.38G > C (p.G13A)	MEK Inhibitor
13-95124	AML	*NRAS; MLL-AFF1 (KMT2A-AFF1)*	c.182A > G (p.Q61R); Fusion	MEK Inhibitor; DOT1L Inhibitor
14-45760	AML	*NRAS*	c.38G > A (p.G13D)	MEK Inhibitor
13-50662	AML	*PTPN11*	c.1508G > T (p.G503V)	MEK Inhibitor
14-15491	AML	*NUP98-NSD1*	Fusion	DOT1L Inhibitor
14-27243	Neuroblastoma	*NRAS*	c.181C > A (p.Q61K)	MEK Inhibitor
14-70449	Rhabdomyosarcoma	*NRAS*	c.181C > A (p.Q61K)	MEK Inhibitor
14-42817	Neuroblastoma	*KRAS*	c.34G > T (p.G12C)	MEK Inhibitor
15-11925^a^	Osteosarcoma	*MYC*	Overexpression	BET Inhibitor
16-74654	Rhabdomyosarcoma	*FGFR4*	c.1582G > T (p.G528C); c.1648G > C (p.V550L)	FGFR4 Inhibitor
15-23518	Rhabdomyosarcoma	*FGFR4*	c.1648G > C (p.V550L); c.1949G > T (p.R650L); Overexpression	FGFR4 Inhibitor
14-37237	Glioblastoma multiforme	*Gain 12q.14.1 involving CDK2*	Copy number	CDK4/6 Inhibitor
15-44470	Medulloblastoma	*PTCH1, SUFU, ZIC3*	Overexpression	SMO Inhibitor
15-10838	Glioma	*H3F3A; FGFR1*	c.83A > T (p.K28M); c.1731C > A (p.N577K)	HDAC Inhibitor
15-27992	Hepatic rhabdoid tumor	*Homozygous deletion of chr 22q11.23 with homozygous deletion of SMARCB1; Loss of expression of SMARCB1*	Copy number; Loss of expression with biallelic deletion	EZH2 Inhibitor
Tier 4 (Published, presented, in press pre-clinical data demonstrating benefit- different tumor type, same gene)				
15-36388	AML	*MLL3 (KMT2C)*	c.2110G > T (p.E704X)	BET Inhibitor
13-72282	T-ALL	*KRAS; JAK1; STAT5B*	c.40G > A (p.V14I); c.3076A > G (p.K1026E); c.2110A > C (p.I704L)	MEK Inhibitor; JAK Inhibitor; BCL2/BCLX-L Inhibitor
15-66870	Adrenocortical carcinoma	*ALK*	c.3436C > A (p.Q1146K)	ALK Inhibitor^b^
Tier 5 (Anything else the molecular tumor board thought was sufficient to qualify for treatment planning)				
15-16072	ALL	*SMARCC2-PDGFRB*	Fusion	TKI
14-75899	Neuroblastoma	*CDK4, MDM2*	Overexpression	NEPENTHE trial [NCT02780128]
15-11925^a^	Osteosarcoma	*MCL1, CCNE1*	Overexpression	CDK4/6 Inhibitor
15-35162	Osteosarcoma	*CUL4A*	Overexpression	NAE Inhibitor
14-71727	Osteosarcoma	*RAD51C*	c.24 T > G (p.F8L)	PARP Inhibitor
15-83826	Osteosarcoma	*PDGFRA, KDR (VEGFR2)*	Overexpression	MTKI^b^
13-21968	Congenital fibrosarcoma	*EML4-NTRK3*	Fusion	ALK Inhibitor
14-84044	Inflammatory myofibroblastic tumor	*VCAN-IL23R*	Fusion	JAK Inhibitor^b^

^a^Same patient

^b^Targeted therapy received

*ALL* acute lymphoblastic leukemia, *AML* acute myeloid leukemia, *CML* chronic myeloid leukemia

Examples of targetable alterations include the identification of a *cKIT* (p.Asn655Lys) [27] mutation in a 7-year-old boy with acute myeloid leukemia (AML), who was subsequently treated with palliative imatinib and achieved a near-complete clearing of peripheral blood leukemic blasts with a sustained response for 9 months. The RNA expression data also led us to identify a *BCR-ABL1-*like [28] expression pattern in a 9-year-old girl with relapsed, refractory B-cell acute lymphoblastic leukemia (ALL). Subsequent analysis identified a *NUP214-ABL1* [29] fusion by real-time polymerase chain reaction (RT-PCR) and the addition of dasatinib to the third-line induction regimen resulted in a deep remission allowing for a curative bone marrow transplant. These results demonstrate the utility of comprehensive genomic characterization to identify clinically actionable alterations in pediatric oncology patients.

### Clinical impact of non-targetable somatic mutations

While many studies have focused on actionable alterations, the potential clinical impact of non-targetable alterations was also evaluated. Genomic alterations identified by sequencing helped to confer a molecular diagnosis in 23 patients and identified prognostic, pharmacogenomic, and other significant health maintenance recommendations in 32 patients (Table 3). Although these findings do not meet the definition of “actionability,” the clinical impact of such findings can be quite profound. For example, the identification of a *STAT5B* mutation [30] in a 5-year-old girl erroneously diagnosed with T-cell ALL helped to establish a diagnosis of gamma-delta T-cell lymphoma. Also, identification of a *PTPN11* mutation in a 4-year-old boy contributed to a change in his diagnosis from de novo AML to juvenile myelomonocytic leukemia (JMML) which evolved into AML [31].

**Table 3:** Clinical utility beyond targetable somatic mutations

PIP ID	Diagnosis	Alteration	Mutation (change)	Clinical utility	Implication
Sequence mutations					
15-63375	AML changed to JMML	*PTPN11; SETB1*	c.181G > T (p.D61Y); c.2602G > A (p.D868N)	Diagnostic [31, 46]	JMML
13-72282^a^	T-ALL	*STAT5B*	c.2110A > C (p.I704L)	Diagnostic [30, 47]	Gamma-delta T-cell lymphoma
15-26188	ALL	*NT5C2*	c.1219G > T (p.D407Y)	Pharmacogenomic [32, 33]	Affects therapy
13-45348	AML	*IDH1*	c.394C > T (p.R132C)	Diagnostic [48, 49]	Maffucci syndrome
14-53198	AML	*CEBPA*	c.939_940insAAG (p.K313_V314insK); c.326_327insC (p.P109fs)	Prognostic [50]	Improved prognosis
15-10838	Glioma	*H3F3A*	c.83A > T (p.K28M)	Prognostic [51]	Poor prognosis
14-37237	Glioblastoma multiforme	*H3F3A*	c.83A > T (p.K28M)	Prognostic [52, 53]	GBM subgroup, K27
14-35585	Renal cell carcinoma	*VHL*	c.497 T > G (p.V166G)	Diagnostic [54]	Von Hippel Lindau
14-47205	Nephroblastomatosis	*PIK3CA*	c.1035 T > A (p.N345K)	Diagnostic [55]	Nephroblastomatosis
14-78154^a^	Medulloblastoma	*KDM6A*	c.2989_2990dupAT (p.M997fs)	Prognostic [56]	Risk stratification, group 4
14-75899	Neuroblastoma	*ATRX*	c.5239delA (p.T1747fs)	Prognostic [57]	Poor prognosis
14-10141	Pleuropulmonaryblastoma	*DICER1*	c.5438A > G (p.E1813G)	Health Maintenance [58]	DICER syndrome
Transcriptome analysis and CNV					
14-24794	AML	*CBFB-MYH11*	Fusion	Prognostic [59]	Low-risk stratification
15-64793	B-ALL	*FOXP1-ABL1*	Fusion	Prognostic [60]	High-risk stratification
15-84578	AMKL	*CBFA2T3-GLIS2*	Fusion	Diagnostic [61]; Prognostic [35]	AMKL; Poor prognosis
14-85546	CML	*BCR-ABL1*	Fusion	Diagnostic [59]	CML
13-72282^a^	T-ALL	*Isochromosome 7q*	Copy number change	Diagnostic [39]	Gamma-delta T-cell lymphoma
15-46387	Rhabdomyosarcoma	*PAX7-FOXO1*	Fusion	Diagnostic [62, 63]; Prognostic [64]	Rhabdomyosarcoma; High-risk group
13-81192	Alveolar soft part sarcoma	*ASPSCR1-TFE3*	Fusion	Diagnostic [65]	Alveolar soft part sarcoma
13-65217	Ewing sarcoma	*EWSR1-FLI1*	Fusion	Diagnostic [66]	Ewing sarcoma
15-47087	Ewing sarcoma	*EWSR1-FLI1; Low expression of PAX8, FHIT, CASP10, CHD2, with high expression of CHD11, FUS, and MTA1*	Fusion; Expression pattern	Diagnostic [66]; Prognostic [36]	Ewing sarcoma; Poor prognosis
13-21968	Undifferentiated sarcoma	*EML4-NTRK3*	Fusion	Diagnostic [34]	Infantile fibrosarcoma
16-88073	Ependymoma	*C11orf95-RELA; Alternating gains and losses on chr 11 and 22, consistent with a “chromothripsis-like” pattern*	Fusion; Copy number change	Prognostic [67]; Diagnostic [68]	Poor prognosis; RELA-type supratentorial ependymoma
14-27243	Neuroblastoma	*MYCN amplified, deletion at 1p and 11q, gain 17q; MYCN over expressed*	Copy number change; Overexpression	Prognostic [69, 70]	Risk-based therapy
14-42817	Neuroblastoma	*MYCN non-amplified, no LOH at 1p11q; MYCN not over expressed*	Copy number change; No overexpression	Prognostic [69, 70]	Risk-based therapy
15-39486	Neuroblastoma	*MYCN non-amplified, no LOH at 1p11q; MYCN not over expressed*	Copy number change; No overexpression	Prognostic [69, 70]	Risk-based therapy
14-44070	Neuroblastoma	*MYCN amplified, loss of 1p, gain of 1q and 17q; MYCN over expressed*	Copy number change; Overexpression	Prognostic [69, 70]	Risk-based therapy
15-88980	Hepatoblastoma	*Amplification of 11q13.2 including CCND1; Over expression of CCND1*	Copy number change; Overexpression	Prognostic [71]	Good prognosis
15-49177	Medulloblastoma	*IMPG2, GABRA5, LAPTM4B, MAB21L2, NPR3, MFAP4, NRL, ZFPM2, TSHZ3, IGF2BP3, GALNT14, GPR98; Loss of 10q22.2-10qter involving PTEN and SUFU, loss of 17p, gain of 17q*	Overexpression; Copy number change	Prognostic [72]	Risk stratification, subgroup 3/4
15-70532	Ependymoma	*Gain of 1q, loss of 6q*	Copy number change	Prognostic [73–75]	Poor prognosis
14-78154^a^	Medulloblastoma	*KCNA1, RBM24, KLHL13, EN2, SNCAIP, PDE1C, GRM8, KCNIP4, EXPH5, UNC5D, NID2, ST18, GPR12, SH3GL3; i17q*	Overexpression; Copy number change	Prognostic [72]	Risk stratification, subgroup 4
15-40141	Pleomorphic xanthoastrocytoma	*Gain of chromosome 7, LOH at 9p*	Copy number change	Diagnostic [76]	Pleomorphic xanthoastrocytoma
15-97336	Small round blue cell tumor	*EWSR1-WT1*	Fusion	Diagnostic [77]	DSRCT
15-34296	Ependymoma	*TNC, CALB1, PLAG1, ALDH1L1, RELN*	Overexpression	Prognostic [37]	Risk stratification, group A, poor prognosis
15-80972	ATRT	*LOH at 22q11.21qter, including SMARCB1; ASCL1*	Copy number change; Overexpression	Diagnostic [78]; Prognostic [79]	ATRT; Improved prognosis

^a^Same patient

*ALL* acute lymphoblastic leukemia, *AMKL* acute megakaryoblastic leukemia, *AML* acute myeloid leukemia, *ATRT* atypical teratoid rhaboid tumor, *CML* chronic myeloid leukemia, *CNV* copy number variation, *JMML* juvenile myelomonocytic leukemia

The identification of resistance alleles likewise is not considered actionable, but may carry significant clinical implications. For example, in the 9-year-old girl with the relapsed *NUP214-ABL1* B-ALL, the finding of a *NT5C2* mutation associated with resistance to nucleoside analog therapies [32, 33] had clear implications for her salvage therapy. In aggregate, sequencing results were clinically informative for diagnostic, prognostic, or pharmacogenomic purposes in 38 patients (38%).

### Clinical impact of transcriptome and CNV analysis beyond target identification

Clinical impact by RNA-seq and CNV analysis was demonstrated in 23/33 patients (70%) (Table 3). Gene fusions confirming diagnosis were found in five patients: *BCR-ABL1* (chronic myeloid leukemia), *ASPSCR1-TFE3* (alveolar soft part sarcoma), *EWSR1-FLI1* in two patients (Ewing sarcoma), and *EWSR1-WTI* (desmoplastic small round cell tumor). A novel *EML4-NTRK3* fusion found in a 2-year-old boy supported a change in diagnosis from undifferentiated sarcoma to infantile fibrosarcoma [15, 34]. In one patient, a *CBFA2T3-GLIS2* [35] fusion confirmed the diagnosis of acute megakaryoblastic leukemia (AMKL), was associated with poor prognosis, and supported the recommendation for a bone marrow transplant. A *PAX7-FOXO1* fusion was diagnostic and prognostic in a toddler with histologically defined solid alveolar rhabdomyosarcoma, but in whom FISH analysis using the FOXO1A (FKHR; 13q14.1) break-apart probe was repeatedly negative.

CNV was inferred from the WES data and relative gene expression was determined by reference to an averaged gene expression model. Segmental and gene expression changes having prognostic implications were identified in 11 patients with a variety of diagnoses. Four patients diagnosed with neuroblastoma could be stratified based on RNA-seq and CNV: one high-risk patient with MYCN amplification, LOH at 1p and 11q, gain of 17q, and MYCN overexpression; one high-risk patient with MYCN amplification, LOH at 1p, gain of 17q, and MYCN overexpression; one high-risk patient without MYCN amplification or LOH at 1p and 11q, and no evidence of MYCN overexpression; and one intermediate-risk patient without MYCN amplification or LOH at 1p and 11q and no evidence of MYCN overexpression. Medulloblastoma subgrouping was supported by overexpression and CNV in two patients. Poor prognostic features were found in two other patients: low expression of *PAX8*, *FHIT*, *CASP10*, *CHD2*, with high expression of *CHD11*, *FUS*, and *MTA1* in a patient with Ewing sarcoma [36], and gain of 1q and loss of 6q and overexpression of *TNC*, *CALB1*, *PLAG1*, *ALDH1L1*, and *RELN* in a patient with ependymoma [37]. Overexpression of *CCND1* in a patient with hepatoblastoma was considered a good prognostic indicator. One patient with AML with a *CBFB-MYH11* fusion could be assigned to risk-based therapy and the diagnosis of gamma-delta T-cell lymphoma [38, 39] was also corroborated by CNV with isochromosome 7q.

### Clinically impactful germline alterations

A total of 90 patients had germline tissue sequenced. Cancer WES included germline analysis in 68/90 patients. Tumor sequencing plus constitutional WES was performed in eight patients and 14 patients had only germline tissue sequenced for a variety of indications including clinical suspicion of cancer predisposition or of an underlying immunologic defect responsible for the development of lymphoma or hemophagocytic lymphohistiocytosis (HLH).

Clinically impactful germline alterations (Table 4) were found in 18/90 patients (20%): 11/57 patients with solid tumors (19%) and 7/33 patients with hematologic conditions (21%). In the solid tumor category, two alterations in *APC* were diagnostic: one in a patient with hepatoblastoma and a family history consistent with familial adenomatous polyposis (FAP; p.R1114) and one associated with newly appreciated Gardner’s syndrome (p.E1554fs) in a 14-year-old boy with pilomatricomas and epidermoid cysts prior to his carcinoma diagnosis. Two variants in *ATM* (p.R189K, p.K2756*) were found in a 16-year-old boy with medulloblastoma inferring an increased risk for developing other cancers. All were referred for genetic counseling and consideration for future cancer screening in the patient and family.

**Table 4:** Clinically impactful germline mutations

PIP ID	Diagnosis	Alteration	Mutation	Change	Mutation type	Clinical utility	Implication	Previously known or suspected
Hematologic conditions								
14-19751	HLH	*MLL2*	c.11640_11640delG; c.15631G > A	p.M3881Cfs*9 *(de novo)*; p.E5211K	Compound heterozygous, frameshift	Diagnostic	Kabuki syndrome; Transplantation withheld	No
15-90485	HLH	*C1QA*	c.622C > T	p.Gln208Ter	Homozygous, nonsense	Diagnostic	C1Q deficiency	No
15-33031	MDS	*GATA2 (de novo)*	c.16delG	p.(E6fs)	Heterozygous, frameshift	Supports transplant recommendation; Genetic counseling for family	Increased risk for developing AML and MDS	No
14-92247	ALL	*PMS2*	c.1376C > G	p.S459X	Homozygous, missense	Diagnostic; Health maintenance/ Genetic counseling	Constitutional mismatch repair deficiency syndrome; Lynch syndrome (parents)	No
15-46877	HLH	*XIAP*	c.1328G > C	p.R443P	Missense	Diagnostic	X-linked lymphoproliferative syndrome 2 (XLP2); Transplantation recommended	No
14-19750	AML	*RUNX1*	c.806-2A > G	r.Spl?	Heterozygous splice site	Diagnostic	Familial platelet disorder; Transplantation donor changed from sibling to unrelated donor	No
Solid tumors								
14-56374	Hepatoblastoma	*APC*	c.3340C > T	p.R1114	Nonsense	Diagnostic; Health maintenance/ Genetic counseling	Familial adenomatous polyposis	Yes
15-33544	Poorly differentiated carcinoma with focal neuroendocrine differentiation	*APC (de novo)*	c.4660_4661insA	p.E1554fs	Frameshift	Diagnostic; Health maintenance/ Genetic counseling	Gardner syndrome; Familial adenomatous polyposis	No
15-35162	Osteosarcoma	*RB1*	c.1216-3A > G	p.?	Splice site	Health maintenance/ Genetic counseling	Increased risk for developing other cancers	Yes
15-44470	Medulloblastoma	*ATM*	c.566G > A; c.8266A > T	p.R189K; p.K2756*	Missense, nonsense	Health maintenance/ Genetic counseling	Increased risk for developing other cancers	No
15-78886^a^	Pineoblastoma	*UGT1A1*	*28 allele (“(TA)7TAA”)		Homozygous	Pharmacogenomic	Drug sensitivity	No
15-78886^a^	Pineoblastoma	*DICER1*	c.4807dupC	p.L1603Pfs	Frameshift	Health maintenance/ Genetic counseling	Risk of ovarian Sertoli-Leydig cell tumor	No
15-17264	Hepatocellular carcinoma	*UGT1A1*	*28 allele		Heterozygous	Pharmacogenomic	Drug sensitivity	No
ACMG secondary findings								
15-29224	AML	*TP53*	c. 644G > A	p.S215N	Missense	Affects therapy; Health maintenance/ Genetic counseling	Explains lack of response to conventional therapy; Increased risk for developing other cancers	No
14-59462	Nested stromal epithelial tumor of the liver	*BRCA1*	c.68._69delAG	p.Glu23Valfs	Frameshift	Health maintenance/ Genetic counseling	Breast cancer	No
16-88073	Ependymoma	*BRCA1*	c.5587_5594delGTAGCACT	p.V1863Lfs*35	Frameshift	Health maintenance/ Genetic counseling	Breast cancer	No
14-75899	Neuroblastoma	*RYR1*	c.6838G > A	p.V2280I	Missense	Health maintenance/ Genetic counseling	Malignant hyperthermia	No
14-13487	Osteosarcoma	*TNNT2*	c.422G > A	p.Arg141Gln	Heterozygous	Health maintenance/ Genetic counseling	Dilated cardiomyopathy	No
15-34296	Ependymoma	*VHL*	c.539 T > C	p.I180T	Missense	Health maintenance/ Genetic counseling	Von Hippel Lindau syndrome	No

^a^Same patient

*ALL* acute lymphoblastic leukemia, *AMKL* acute megakaryoblastic leukemia, *AML* acute myeloid leukemia, *HLH* hemophagocytic lymphohistiocytosis, *JMML* juvenile myelomonocytic leukemia

In patients with hematologic conditions, the incidence of germline alterations related to the primary diagnosis was observed in five patients (15%). A homozygous pathogenic variant in *C1QA* (p.Gln208Ter) diagnostic of C1Q deficiency was identified in a 2-year-old girl with HLH. A homozygous pathogenic variant in *PMS2* (p.S459X) diagnostic of congenital mismatch repair deficiency was identified in one patient with T-cell lymphoblastic lymphoma and consanguineous parentage [40]. A likely pathogenic variant in *XIAP* (p.R443P) was identified in a 6-year-old girl with HLH, recurrent EBV infections, and suspected underlying immunodeficiency. Germline testing also revealed a heterozygous pathogenic splicing variant in *RUNX1* (c.806-2A > G, r.Spl) in a patient with AML referred for transplantation for persistent thrombocytopenia following chemotherapy [41]. Both an HLA-matched sibling with borderline low platelets and the father were found to carry the same variant. An unrelated donor source was selected. A 2-month-old patient hospitalized for fulminant hemophagocytic syndrome was referred for evaluation of presumed familial HLH and was considered for hematopoietic stem cell transplantation. However, germline WES identified a pathogenic homozygous mutation in *MLL2* (p.M3881Cfs*9) establishing the diagnosis of Kabuki syndrome [42] and familial HLH was ruled out due to the lack of alterations in any HLH-associated genes and subsequently plans for a bone marrow transplant were averted.

ACMG secondary findings were identified in six patients (Table 4) and were returned to the families by clinical genetics. A germline *BRCA1* mutation was discovered in an 18-year-old boy with a rare hepatic tumor and a 17-year-old girl with ependymoma. A *TP53* mutation was found in a 1-year-old girl with AML, a *TNNT2* mutation associated with dilated cardiomyopathy was found in a 15-year-old boy with osteosarcoma, a *RYR1* mutation associated with malignant hyperthermia was found in a 7-year-old girl with neuroblastoma, and a mutation in *VHL* was found in a 2-year-old boy with ependymoma.

Germline variants classified as VOUS (Additional file 5: Table S6) were not returned to patients except if they met the following criteria: (1) the variant was predicted to be destructive; (2) the variant was in a well validated cancer-associated gene; and (3) a second somatic alteration was identified or the variant was reduced to homozygosity in the tumor. Clinical genetics returned a VOUS to four patient families meeting these criteria, including an *ITK* (p.V175V) mutation in a 7-year-old girl with Hodgkin lymphoma and Epstein-Barr virus, an *SDHC* (p.G75D) mutation was found in a 12-year-old boy with ALL, a *DICER1* (p.D609Y) mutation in an 18-year-old boy with ALCL, and an *APC* (p.V1822D) mutation in a 7-year-old boy with Ewing sarcoma.

### Clinical impact of WES

**Fig. 4:**
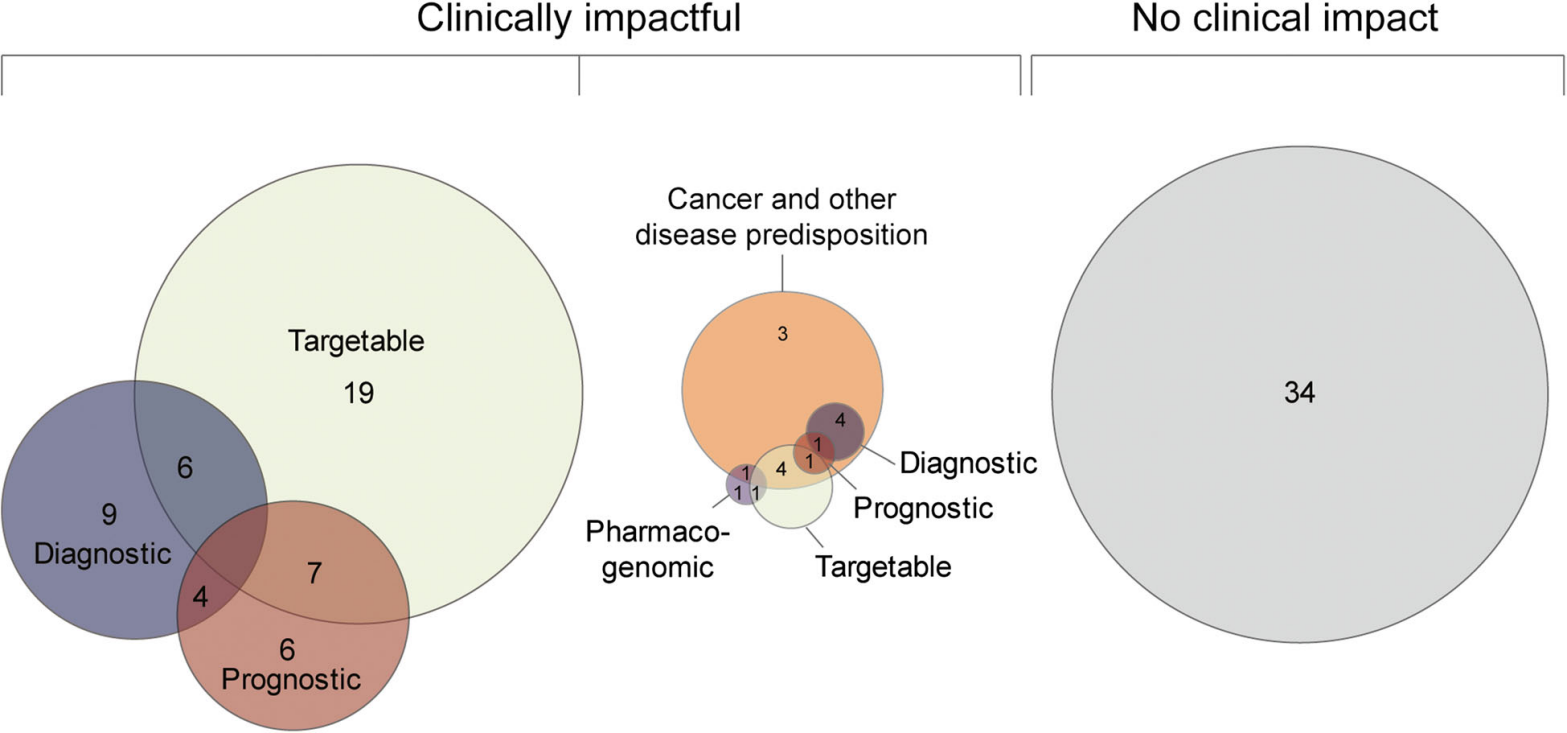
Clinically impactful results. The PIPseq experience yielded clinically impactful results in 67/101 cases. The *Venn diagrams* depict the complexity of overlapping findings within patients. That is, a patient may have a single finding fitting more than one category, whereas another patient may have a finding fitting one category and another finding fitting a different category. For example, results categorized as Targetable/ Diagnostic (n = 6) are as follows: BCR-ABL1; IDH1; PIK3CA; EML4-NTRK3; [STAT5B, KRAS, JAK1/ STAT5B, i7q]; and [TMEM106B-BRAF/ gain chr 7, LOH 9p], with non-bracketed results representing a single finding fitting two categories and results within brackets representing those that were Targetable/ Diagnostic, respectively. Similarly, results categorized as Targetable/ Prognostic (n = 7) are as follows: FOXP1-ABL1; [TET2/ CEBPA]; [H3F3A, FGFR1/ H3F3A]; [NRAS/ MYCN amp, del 1p and 11q, gain 17q]; [c-KIT, TET2, FLT3, NRAS/ CBFB-MYH11]; [KRAS/ No LOH 1p11q]; and [Gain 12q.14.1 involving CDK2/ H3F3A]. Individual patient results are provided in Tables 2, 3, and 4

To determine the overall clinical impact of NGS cancer analysis, we evaluated each case as to whether the sequencing data were of potential utility to the referring physician in a clinically meaningful manner. Overall, clinically impactful results were found in 67/101 cases (66%) (Fig. 4). Potentially actionable alterations were found in 38% of cases. In 23% of the cases, the data obtained provided diagnostic significance. Importantly, germline predisposition to cancer was identified in 14% of all cases.

**Fig. 5:**
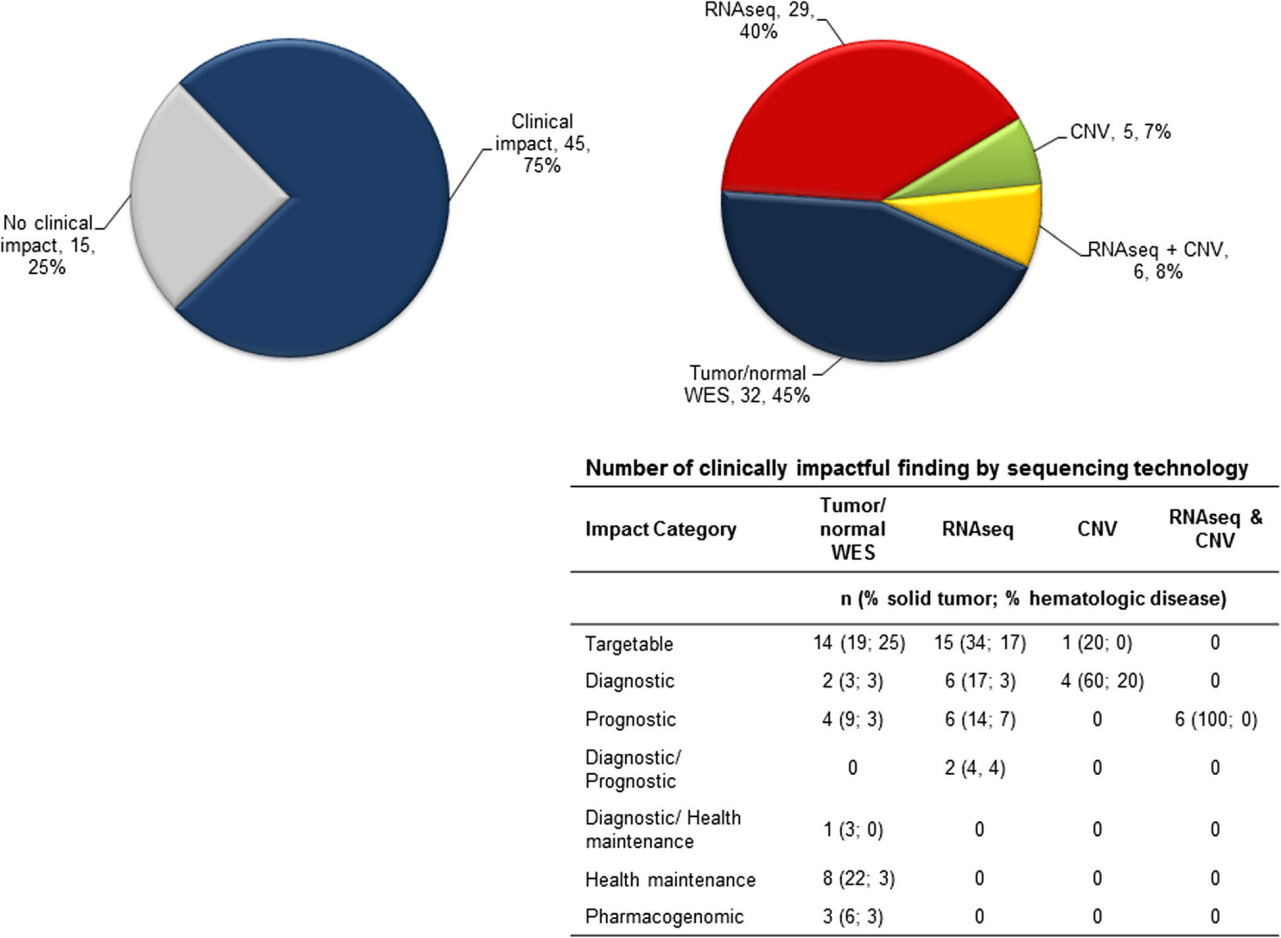
Clinical impact of WES and RNA-seq by sequencing technology. Sixty patients had full tumor/normal WES (including CNV) and RNA-seq (cWES) performed. A total of 72 clinically impactful results were found in 45/60 cases (75%). A *pie chart* of the overall clinical impact of cWES is presented on the *left* with a *pie chart* and *table* showing the number of impactful findings by sequencing technology on the *right*. For six patients, CNV and overexpression together yielded prognostic information in four patients with neuroblastoma and two patients with medulloblastoma

WES and RNA-seq allows for significant additional analytical endpoints (CNV, fusions, gene expression) over targeted gene panels. Focusing on the 60 cases with full tumor/normal WES and RNA-seq (cWES), the resulting data were clinically impactful in 45 cases (75%) (Fig. 5). A total of 72 potentially clinically impactful results were found with cWES accounting for 85% of the findings (tumor/normal WES: 45%, *n* = 32; RNA-seq: 40%, *n* = 29) followed by CNV (7%, *n* = 5) and RNA-seq and CNV together in 8% (*n* = 6). Of the 30 potentially targetable aberrations found, 14 were by tumor/normal WES, 15 by RNA-seq, and one by CNV (Fig. 5).

## Discussion

In this report, we reviewed the results of the first 101 patients evaluated in our precision cancer medicine program. While we used a variety of analytical approaches matched to the clinical indications, we primarily utilized a combination of tumor/normal WES and tumor RNA-seq. This platform provided several advantages over targeted cancer gene panels, including the ability to identify translocations, segmental chromosomal changes, and relative gene expression changes.

Similar to other sequencing efforts in pediatric oncology, we found that the overall mutational load in our patients was relatively low by comparison to adult cancers [38]. Of significance, we identified germline alterations that predispose to cancer in 14% of our patients. This is slightly higher than other studies that have demonstrated approximately 8.5–10% frequency of germline risk alleles in pediatric oncology patients and may reflect a selection bias to sequence patients with high-risk cancers [12, 43, 44]. These results underscore the need to routinely incorporate germline analysis for pediatric oncology patients.

Although there is a paucity of Tier 1 actionable alterations in pediatric cancers, using a more lenient definition of actionable which includes same gene–different tumor type, likely pathogenic VOUS, and assessment of both clinical and preclinical data, resulted in the identification of potentially actionable alterations in 38% of all patients. This is comparable to other studies and may in itself be sufficient justification for comprehensive genomic analysis in cancer patients [10, 12, 15, 45]. Despite this finding, only 16% of patients received matched targeted therapy. The ability to intervene with targeted therapies is particularly challenging for pediatric patients. Many newer drugs lack efficacy data in pediatric diseases or safety data in children and are therefore not yet approved for administration. Additionally, insurance companies are not obligated to provide coverage for the off-label use of these high-cost agents. Compassionate use experimental therapies undergoing clinical testing or recently approved agents for adults are also rarely granted for pediatric patients. Finally, a number of targeted agents are not anticipated to have single-agent efficacy (e.g. MEK inhibition for *RAS* mutant tumors). Together, the lack of pediatric experience and opportunities with combination therapy represent additional constraints in pediatric oncology.

Nevertheless, we believe that narrowing the definition of benefit to the identification of actionable targets and matched targeted therapy underestimates the potential clinical utility of comprehensive genomic analysis. We provide examples of genomic alterations that are not actionable per se, but which have significant clinical impact including for diagnostic, prognostic, or pharmacogenomics purposes. Taking a broad view of clinical impact, it is notable that the data from our sequencing platform impacted clinical decision-making in over two-thirds of all cases. With the increase in genomic medicine programs and the growing body of knowledge, the adoption of a more inclusive definition of clinical utility that does not narrowly focus on drug selection for patients with a specific biomarker is an important point to consider when incorporating NGS technologies into clinical practice.

Most cancer sequencing programs focus on interrogation of tumor DNA. It is notable that in our program the transcriptome data were responsible for a number of clinically impactful calls that were not evident from interrogating the DNA alone. In addition to verifying variants identified in the DNA analysis, the transcriptome was used to identify translocations and was mined to identify signaling pathway activity. We generated a model from transcriptomes in our database, allowing us to identify expression outliers. We were also able to project the gene expression data into existing gene expression datasets for classification purposes, allowing us for example, to identify a *BCR-ABL1*-like gene expression pattern. Therefore, assessing tumor RNA is an important component of comprehensive genomic approaches and in our series samples interrogated with both WES and RNA-seq characterization resulted in clinically impactful data in 75% of cases.

The importance of assessing germline in addition to cancer DNA is evident from the 14% incidence of germline variants that may predispose to cancer. These findings clearly have broad implications that impact not only the patient but potentially the entire family. Moreover, the identification of germline risk offers opportunities for prevention and early screening and detection. It is notable that given the opportunity to opt out of this knowledge, nearly all families actively choose for the return of these results, underscoring the fallacy of the paternalistic view that families need to be protected from learning these findings.

Finally, extending beyond a fuller appreciation for the potential clinical impact of sequencing technologies, it is important to consider that genomic approaches do not just provide incremental data, but may replace many conventional tests. Currently, many genetic alterations can be identified by standard approaches, such as karyotype and FISH, and with faster turnaround times. Similarly, existing NGS panels, which allow the detection of mutations and/or fusions of clear clinical relevance, may be adequate in certain clinical scenarios. Nevertheless, in an era where initial diagnostic biopsies are often performed through minimally invasive approaches, there is a compelling argument to utilize comprehensive approaches with minimal tissue requirements. As the cost of NGS declines, the ability to comprehensively interrogate the genome may supersede the need for sequential, potentially tissue-exhausting directed testing, with the added benefit of uncovering rare targetable and potentially unexpected genomic drivers.

## Conclusions

Our results demonstrate the feasibility of incorporating clinical NGS into pediatric hematology-oncology practice. While the frequency of finding actionable alterations is consistent with reports of other pediatric oncology sequencing endeavors [10, 12, 15, 45], we feel this singular attribute grossly underestimates the potential clinical utility of these data. The ability to avoid ineffective/inappropriate therapies, to solidify a definitive diagnosis, and to identify pharmacogenomics modifiers all have clinical impact. Taking this more inclusive view, it is striking that the sequencing data were found to be clinically impactful in 66% of all cases tested through our program and in 75% of cases comprehensively assessed using cWES and RNA-seq. The value proposition for next generation diagnostics, therefore, should be measured both on the clinical impact of the data and the ability to replace multiple conventional single endpoint assays with a single comprehensive view of the genome.

## Additional files

Additional file 1: A PDF file containing Supplementary Methods. Supplementary Methods DNA and RNA extraction; somatic variant calling strategy; CNV; transcriptome analysis. (PDF 82 kb)
Additional file 2: A PDF file containing Supplementary Tables S1–S5. **Table S1** Quality statistics for WES. **Table S2** Accession ID for all genes and fusions referenced in the manuscript. **Table S3** Individual diagnoses. **Table S4** Cancer WES consent preferences. **Table S5** Reported somatic alterations by tier and germline alterations by category. (PDF 240 kb)
Additional file 3: De-identified clinical cWES report. (PDF 157 kb)
Additional file 4: A TIFF file containing Supplementary Figure S1. **Figure S1** Total somatic mutation load across all patients. The total mutational load across patients was 19,308 variants (mean, 216.9; SD, 829.3; median, 69). By diagnostic category, the total mutational load for solid tumors was 5853 variants (mean, 97.5; SD, 111.7; median, 86; range, 15–881), and for hematologic conditions was 13,455 variants (mean, 463.9; SD, 1428.8; median, 52; range, 14–5950). (TIF 845 kb)
Additional file 5: An excel file containing Supplementary Table S6. **Table S6** Somatic and germline variants of unknown significance. (XLSX 20 kb)

## Abbreviations

ACMG: American College of Medical Genetics
CNV: copy number variation
cWES: cancer whole exome sequencing
EMR: electronic medical record
FFPE: formalin fixed paraffin embedded
HLA: human leukocyte antigen
HLH: hemophagocytic lymphohistiocytosis
VOUS: variants of uncertain significance
WES: whole exome sequencing
