# Supplementary material for: Implementation of next generation sequencing into pediatric hematology-oncology practice: moving beyond actionable alterations
Source: Genome Med. 2016 Dec 23;8:133. doi: 10.1186/s13073-016-0389-6 (PMC5180407; doi:10.1186/s13073-016-0389-6)
Supplement: Additional file 1: — A PDF file containing Supplementary Methods. Supplementary Methods DNA and RNA extraction; somatic variant calling strategy; CNV; transcriptome analysis. (PDF 82 kb) [file 13073_2016_389_MOESM1_ESM.pdf]

## Supplementary Methods

### DNA and RNA Extraction:

Samples were evaluated by a pathologist to ensure that there was at least 50% tumor content and that blood/bone marrow tumor specimens had at least 50% cancer cells by flow cytometry performed on a concomitant sample. DNA from macro-dissected paraffin embedded tumor, OCT-embedded frozen tissue, bone marrow, peripheral whole blood, or buccal swabs was extracted using the Qiagen Qiamp tissue kit (tissue); Qiagen Qiasymphony DNA mini kit (blood and bone marrow); or Qiagen DNA micro kit (buccal swabs). RNA was extracted using the Qiagen RNeasy (fresh, frozen) or Qiagen RNeasy FFPE (paraffin embedded tissue). Prioritization of testing in cases of limited sample was made jointly by the clinician and molecular pathologist.

### Somatic Variant Calling Strategy:

Because this is a clinical assay, validated primarily for pediatric cancer cases which tend to have a low mutation burden, we deliberately chose an approach that would maximize the number variants output, to minimize the likelihood of filtering out actionable mutations, combined with a conservative approach to variant prioritization for reporting only high quality variants with a high likelihood of being drivers or clinically significant with the understanding that the raw data may be re-analyzed at any time (and available for re-analysis by others at the request of patients and their authorized treating practitioners). To compensate for variability in coverage in tumor and germline samples, we use a 5% cut-off for germline variants and 10% cut-off for variants in tumor (this partially explains the higher number of SNVs in tumor than normal samples; the other explanation is LOH/chromosomal monosomies with loss of heterozygous germline variants in

samples with a very high tumor fraction). Prior to filtration and visual review of pile-ups by a pathologist, 93% of called somatic variants in regions with 100X coverage or greater in tumor were reproducible in internal reproducibility validation of three samples; for regions with less than 51-100X coverage in tumor, reproducibility was 57%, and in regions with less than 50X coverage in tumor, it was 18%. However, all variants of interest are reviewed by at least two individuals, with pile-ups of tumor and normal. Only variants that are seen on multiple independent fragments (at least two), with read-balance ratios in the same "direction" as the wild type, and not seen on well-covered (at least 20X) normal, and that cannot be excluded as likely benign are reported. In the same samples used for reproducibility analysis, all 61 such variants were 100% reproducible in three independent runs.

**Copy Number Variation:**

In chromosomes or chromosomal regions with copy number alterations or copy number neutral LOH, heterozygous germline variants showed VAFs in tumor specimens that reflected the weighted average of the tumor-specific VAF and the 50% VAF in contaminating normal nuclei, resulting in two tracks of VAFs. For example, in a tumor sample with 50% tumor, single copy loss of a chromosomal region results in VAFs of 0.33 and 0.67 and copy number neutral LOH, and VAFs of 0.25 and 0.75, with and without loss on EXCAVATOR analysis, respectively.

**Transcriptome Analysis:**

The relative expression of each gene (log2 ratio relative to sample-specific median for the 8000 ubiquitously expressed genes) was ranked across the 124 normal samples. For each clinical sample, this ratio was compared to the 124 normal samples and its percentile rank determined.

FPKMs, log-ratios and percentile ranks were output for genes in the top 10 and bottom 10 percentiles, and in a separate worksheet, for all genes. To confirm that our database of normal tissues was representative, we reproduced our predictions with 2921 normal transcriptomes from the GTEx database, v.4 (http://gtexportal.org/home/datasets). Lastly, many if not most of the differentially expressed genes were expected to be lineage specific for a given tumor. Therefore, these data were made available to the evaluating physician to answer specific questions (e.g., interrogating genes with high/low expression patterns characteristic of medulloblastoma subtypes; genes with high/low expression in ABL1 subtype), or for re-analysis by laboratories with larger datasets of a specific tumor type for sub-classification of the tumor type (e.g., as done with ALL case to identify ABL1 expression pattern).
