# Supplementary material for: Implementation of next generation sequencing into pediatric hematology-oncology practice: moving beyond actionable alterations
Source: Genome Med. 2016 Dec 23;8:133. doi: 10.1186/s13073-016-0389-6 (PMC5180407; doi:10.1186/s13073-016-0389-6)
Supplement: Additional file 2: — A PDF file containing Supplementary Tables S1–S5. Table S1 Quality statistics for WES. Table S2 Accession ID for all genes and fusions referenced in the manuscript. Table S3 Individual diagnoses. Table S4 Cancer WES consent preferences. Table S5 Reported somatic alterations by tier and germline alterations by category. (PDF 240 kb) [file 13073_2016_389_MOESM2_ESM.pdf]

Supplementary Table S1. Quality statistics for whole exome sequencing

	Tumor (n=61)				Normal (n=56)			
	avg	sd	p10th	p90th	avg	sd	p10th	p90th
Total_Reads	185145071.33	42119867.17	136769290.66	245894116.09	185322492.14	40633523.52	138966823.14	242943180.23
Aligned_Reads	179408670.64	40912544.21	133993241.15	234519093.94	181549619.94	38725905.77	136734826.67	236868598.77
Reads_Ambiguous_Aligned[a]	178661446.07	40937016.14	133281316.59	233603390.28	180858499.54	38721534.20	136155692.71	236172560.71
Reads_on_Target	146271603.79	34890021.61	106776549.81	194126616.28	148373567.31	32863490.91	110609763.76	192538876.43
Maximum_Coverage	9799.42	6446.57	4256.04	17997.58	9015.24	4436.57	4797.75	16230.59
Average_Coverage	170.06	40.79	123.80	225.76	172.72	38.48	128.28	221.78
ROI_Percent_Covered	98.48	1.21	98.03	99.17	98.72	0.41	98.14	99.12
indel_count	921.47	107.91	792.93	1052.29	1113.98	111.37	974.12	1247.31
snp_count	25403.53	2160.22	23122.77	28484.78	29241.25	2969.76	25902.53	33269.77
N_Ts	18574.38	1426.36	16989.64	20980.78	20300.27	1425.00	18996.78	22835.07
N_Tv	6816.38	827.89	5755.77	8010.96	8948.61	1937.59	6689.01	11081.99
Ts/Tv	2.77	0.18	2.58	3.00	2.45	0.35	2.21	2.87
Het	14275.33	2310.55	11106.68	17176.98	15400.95	1736.32	13701.07	18119.52
Hom	8768.13	786.81	8071.90	10070.65	8727.38	422.59	8383.09	9308.46
Hom/Het	0.64	0.16	0.48	0.90	0.57	0.08	0.48	0.65
LowConf	3229.78	955.33	2157.89	4430.96	6061.80	2199.34	3624.00	7905.16
LowConf/Het	0.23	0.09	0.15	0.34	0.40	0.14	0.24	0.52

Legend:

ROI= region of interest

N_Ts= number of transition variants

N_Tv= number of transversions

Ts/Tv= transition to transvesion ratio

Het= heterozygous variant

Hom= homozygous variant

[a]Output with “Allowable Ambiguous Alignments set to ‘0’”. It is the number of reads that mapped to a unique genomic loci.

Supplemental Table S1 continued

Quality Control/ Assurance:

For whole exome sequencing, the uniformity of coverage is provided by the ROI percent covered, for which the requirement is 98% coverage. At this level of coverage, our analysis showed 10X coverage of 98.5%, and 30X coverage of 94.5% of the region of interest (180 million reads, which is our average). For whole exome, coverage of individual mutations is not output. For the targeted assay, at 500X, over 98% of the ROI is covered at least at 50X.

Supplementary Table S2. Accession ID for the 40 Genes and 16 fusions referenced in the manuscript tables

Gene	Transcript ID	Source
ALK	NM_004304.4	RefSeq
APC	NM_000038.5	RefSeq
ATM	NM_000051.3	RefSeq
ATRX	NM_000489.3	RefSeq
BRCA1	NM_007300.3	RefSeq
C1QA	NM_015991.2	RefSeq
CEBPA	NM_004364.3	RefSeq
KIT	NM_000222.2; NM_00109372.1	RefSeq
DICER1	NM_030621.3	RefSeq
FGFR1	NM_001174067.1	RefSeq
FGFR4	NM_002011.3	RefSeq
FLT3	NM_004119.2	RefSeq
GATA2	NM_001145661.1	RefSeq
H3F3A	NM_002107.4	RefSeq
IDH1	NM_005896.2	RefSeq
JAK1	NM_002227.2	RefSeq
JAK3	NM_000215.3	RefSeq
KDM6A	NM_021140.2	RefSeq
KRAS	NM_033360.2	RefSeq
MLL2	NM_003482	RefSeq

Gene	Transcript ID	Source
MLL3 (KMT2C)	NM_170606.2	RefSeq
NRAS	NM_002524.4	RefSeq
NT5C2	NM_001134373.2	RefSeq
PIK3CA	NM_006218.2	RefSeq
PMS2	NM_000535.5	RefSeq
PTPN11	NM_002834.3	RefSeq
RAD51C	NM_058216.1	RefSeq
RB1	NM_000321.2	RefSeq
RUNX1	NM_001754.4	RefSeq
RYR1	NM_000540.2	RefSeq
SETB1	NM_015559.2	RefSeq
SMARCA4	NM_001128849.1	RefSeq
STAT5B	NM_012448.3	RefSeq
TET2	NM_001127208.2	RefSeq
TNNT2	NM_001001430.1	RefSeq
TP53	NM_000546.4	RefSeq
TSC1	NM_000368.4	RefSeq
UGT1A1	NM_000463.2 c.-53_-52[6,7]	HGVS designation
VHL	NM_000551.3	RefSeq
XIAP	NM_001167.3	RefSeq

Fusion	Transcript ID	Source
ASPSCR1-TFE3	ASPSCR1: NM_024083;<br>TFE3: NM_006521	RefSeq
BCR-ABL1	BCR: NM_004327.3;<br>ABL1: NM_007313.2	RefSeq
C11orf95-RELA	C11orf95: NM_001144936;<br>RELA: NM_001145138, NM_021975	RefSeq
CBFA2T3-GLIS2	CBFA2T3: NM_005187;<br>GLIS2: NM_032575	RefSeq
CBFB-MYH11	CBFB: NM_022845;<br>MYH11: NM_022844	RefSeq
EML4-NTRK3	EML4: NM_191063.3;<br>NTRK3: NM_001012338.2	RefSeq
EWSR1-FLI1	EWSR1: NM_013986.3;<br>FLI1: NM_000217	RefSeq
EWSR1-FLI1	EWSR1: NM_005243;<br>FLI1: NM_002017	RefSeq
EWSR1-WT1	EWSR1: NM_005243;<br>WT1: NM_000378	RefSeq
FOXP1-ABL1	FOXP1: NM_032682;	RefSeq

	ABL1: NM_007313, NM_005157	
MLL-AFF1 (KMT2A-AFF1)	MLL (KMT2A): NM_005933<br>AFF1: NM_005935	RefSeq
NUP98-NSD1	NUP98: NM_016320;<br>NSD1: NM_172349	RefSeq
PAX7-FOXO1	PAX7: NM_013945, NM_001135254, NM_002584;<br>FOXO1: NM_002015	RefSeq
SMARCC2-PDGFRB	SMARCC2: NM_003075;<br>PDGFRB: NM_002609	RefSeq
TMEM106B-BRAF	TMEM106B: NM_001134232, NM_018374;<br>BRAF: NM_004333	RefSeq
VCAN-IL23R	VCAN: NM_004385.4;<br>IL23R: NM_144701.2	RefSeq

Supplementary Table S3. Individual Diagnoses

Diagnosis	N
**Adrenal**	**9**
Neuroblastoma	8
Adrenocortical carcinoma	1
**Sarcoma**	**16**
Osteosarcoma	6
Rhabdomyosarcoma	5
Ewing sarcoma/ PNET	3
Alveolar soft part sarcoma	1
Infantile fibrosarcoma	1
**Renal**	
Wilms tumor	3
Nephroblastomatosis	1
Renal cell carcinoma	1
**Germ Cell**	**4**
Malignant germ cell tumor	2
Immature teratoma	1
PNET transformed from NSMGCT	1
**Hepatic**	**8**
Hepatoblastoma	3
Malignant rhabdoid tumor	2
Hepatocellular carcinoma	2
Nested stromal epithelial tumor of the liver	1
**Brain**	**17**
Medulloblastoma	3
Glioma- NOS	3
Astrocytoma	2
Ependymoma	3
Glioblastoma multiforme	1
Diffuse intrinsic pontine glioma	1
Choroid plexus papilloma	1
Pineoblastoma	1
AT/RT	1
Small round blue cell tumor	1
**Lymphoid**	**17**
ALL	10
Lymphoma	6
Hodgkin lymphoma-like PTLD	1
**Myeloid**	**15**
AML	13
CML	1
MDS	1

**Other- Solid tumors**	**6**
IMT	1
Pilomatrix carcinoma	1
Pleuropulmonaryblastoma	1
Plexiform schwannoma	1
Plexiform fibrohistocytic tumor	1
Mucoepidermoid carcinoma	1
**Other- Hematologic conditions**	**4**
Hemophagocytic lymphohistiocytosis	3
Common variable immune deficiency	1

Supplementary Table S4. Cancer Whole-Exome Sequencing Consent Preferences (n = 67)

	No. (%) by Preference	
	**Yes**	**No**
Clinical		
Return secondary findings	63 (94)	4 (6)
Secondary findings in medical record	46 (69)	21 (31)
Store left over samples	67 (100)	0 (0)
Future contact	66 (98)	1 (2)
Research		
Use leftover samples	65 (97)	2 (3)
Store raw sequencing data	66 (98)	1 (2)
Store raw sequencing data with identifiers	59 (88)	7 (10)
Future contact	62 (93)	5 (7)

Supplementary Table S5. Reported Somatic Alterations by Tier and Germline Alterations by Category

	No. (%) by Diagnostic Category	
	**Solid Tumors**	**Hematologic Conditions**
Somatic		
Tier 1: Known tumor type-specific actionable somatic mutations	0 (0)	0 (0)
Tier 2: Somatic mutations actionable in other tumor types, in targetable pathways, or in well-established cancer genes	32 (32)	36 (45)
Tier 3: Other somatic mutations in cancer genes	6 (6)	5 (6)
Tier 4: Somatic variants of uncertain significance	49 (49)	26 (33)
Germline		
Secondary (ACMG) variant	5 (5)	0 (0)
Secondary (ACMG) variant of uncertain significance	0 (0)	1 (1)
Pathogenic (non-ACMG variant)	3 (3)	7 (9)
Variant affecting therapy (pharmacogenomic)	2 (2)	1 (1)
Variant related to tumor	1 (1)	0 (0)
Variant of uncertain significance	2 (2)	4 (5)
