# Supplementary material for: Implementation of next generation sequencing into pediatric hematology-oncology practice: moving beyond actionable alterations
Source: Genome Med. 2016 Dec 23;8:133. doi: 10.1186/s13073-016-0389-6 (PMC5180407; doi:10.1186/s13073-016-0389-6)
Supplement: Additional file 3: — De-identified clinical cWES report. (PDF 157 kb) [file 13073_2016_389_MOESM3_ESM.pdf]

COLUMBIA UNIVERSITY LABORATORIES

Columbia University Laboratory of Personalized Genomic Medicine

630 West 168th Street New York, New York 10032
212-305-9706, FAX# 212-342-0420

Name:	Case No.: MOP
MRN:	Date Obtained:
D.O.B.	Date Received:
Sex:	Physician:
Location:	Copy to:

MOLECULAR ONCOLOGY

MOLECULAR DIAGNOSTIC TEST:
Cancer Whole Exome Sequencing with Transcriptome

CLINICAL INFORMATION:

SPECIMEN:

TEST RESULTS:

Summary:

EXOME SEQUENCING

Tier 1 (actionable somatic mutations associated with tumor type):

Gene:
Variant:
Variant allelic fraction:
Comment:
Reference:

Tier 2 (somatic mutations in targeted pathways; actionable somatic mutations in other tumor types; somatic mutations in well-established cancer genes):

Gene:
Variant:
Variant allelic fraction:
Comment:
Reference:

Tier 3 (other somatic mutations in cancer genes):

Gene:
Variant:
Variant allelic fraction:
Comment:
Reference:

Tier 4 (somatic variants of uncertain significance):

Gene:
Variant:
Variant allelic fraction:
Comment:
Reference:

Germline variants affecting therapy:

Gene:
Variant:
Variant allelic fraction:
Comment:
Reference:

Secondary germline variants:

Gene:
Variant:
Variant allelic fraction:
Comment:
Reference:

Copy number changes:

Transcriptome sequencing:
1. Fusions:
2. Differential expression outliers:

TEST INFORMATION:

Cancer Whole Exome Sequencing with Transcriptome:
Whole exome sequence is obtained from DNA purified from buccal swab or peripheral blood and the tumor using Agilent Sureselect capture and Illumina HiSeq2500 sequencing. Samples have an average coverage of at least 150-fold, and at least 10 fold coverage of greater than 98% of the region of interest. In addition, transcriptome sequencing is performed on tumor (and when available from normal tissue corresponding to the tumor type). Tumor transcriptomes have at least 50 million independent mappable reads. Results are evaluated for variants in the tumor not present in normal tissue (“somatic variants”). In addition, the normal DNA is evaluated for variants in a set of recommended genes, for which potential interventions are available; and for variants in genes that might affect therapy (“pharmacogenetic variants”).

PURPOSE: To identify driver mutations in the tumor as well as mutations that may modify therapy or allow the patient to enter ongoing clinical trials.

Comments and Limitations:

1. Whole exome sequencing can identify mutations that are currently known to cause disease and mutations that disrupt genes that are known to be associated with disease; some mutations that are not currently known to be pathogenic will not be reported.
2. The list of genes that are reported upon is available upon request.
3. Due to the technology employed in this test, some regions of the exome might not be reported upon due to technical limitations of sequence capture.
4. Structural genomic DNA rearrangements are not currently reported based on data obtained using this methodology. This test was developed and its performance characteristics determined by the Molecular Pathology Laboratory of Columbia University. It has not been cleared or approved by the U.S. Food and Drug Administration. The FDA has determined that such clearance or approval is not necessary.
