# Supplementary figures and images for: Implementation of next generation sequencing into pediatric hematology-oncology practice: moving beyond actionable alterations
Source: Genome Med. 2016 Dec 23;8:133. doi: 10.1186/s13073-016-0389-6 (PMC5180407; doi:10.1186/s13073-016-0389-6)

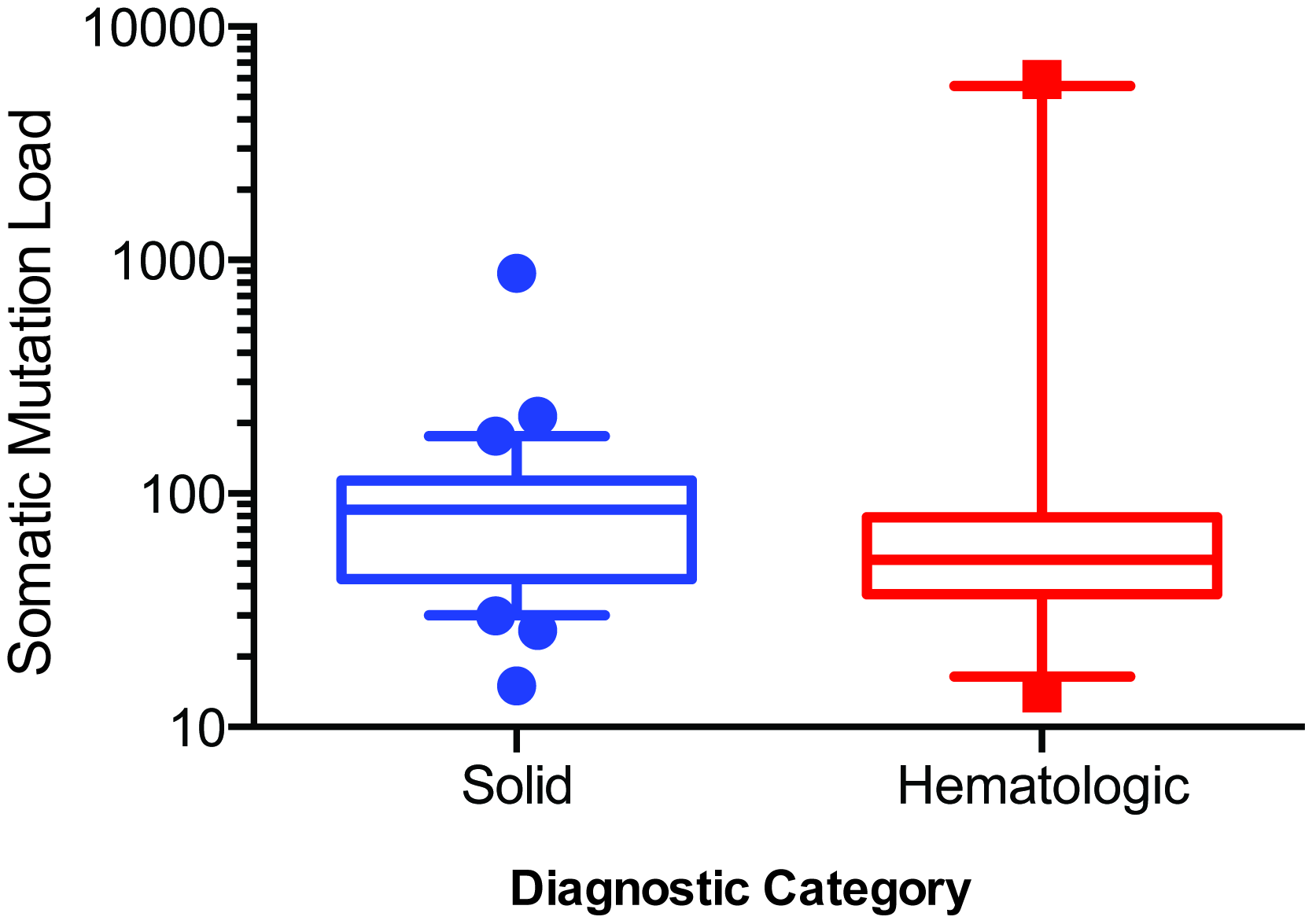

Supplement: Additional file 4: — A TIFF file containing Supplementary Figure S1. Figure S1 Total somatic mutation load across all patients. The total mutational load across patients was 19,308 variants (mean, 216.9; SD, 829.3; median, 69). By diagnostic category, the total mutational load for solid tumors was 5853 variants (mean, 97.5; SD, 111.7; median, 86; range, 15–881), and for hematologic conditions was 13,455 variants (mean, 463.9; SD, 1428.8; median, 52; range, 14–5950). (TIF 845 kb) [file 13073_2016_389_MOESM4_ESM.tif]
